# Supplementary material for: Influenza vaccine effectiveness against medically attended outpatient illness, United States, 2023–24 season
Source: medRxiv. 2024 Oct 30:2024.10.29.24316377. Preprint. [Version 1] doi: 10.1101/2024.10.29.24316377 (PMC11581083; doi:10.1101/2024.10.29.24316377)
Supplement: Supplement 1 [file media-1.docx]

**Supplemental Table 1.** Enrollment dates included by site

| **State** | **Enrollment Institutions** | **Enrollment start date** | **Enrollment end date** |
| --- | --- | --- | --- |
| Arizona | Arizona State University,  Valleywise Health,  Phoenix Children’s Hospital | October 23, 2023 | April 30, 2024 |
| Michigan | University of Michigan,  Henry Ford Health | December 1, 2023 | April 25, 2024 |
| Missouri | Barnes Jewish Memorial Hospital,  St. Louis Children’s Hospital | October 10, 2023 | April 30, 2024 |
| Ohio | University Hospitals at Cleveland,  Cleveland Veterans’ Health Administration | November 27, 2023 | April 11, 2024 |
| Pennsylvania | University of Pittsburgh Medical Center,  Children’s Hospital of Pittsburgh | November 8, 2023 | April 25, 2024 |
| Texas | Baylor Scott & White Health | October 3, 2023 | April 30, 2024 |
| Washington | Kaiser Permanente Washington | October 11, 2023 | April 29, 2024 |

**Supplemental Figure 1.** Exclusion criteria figure
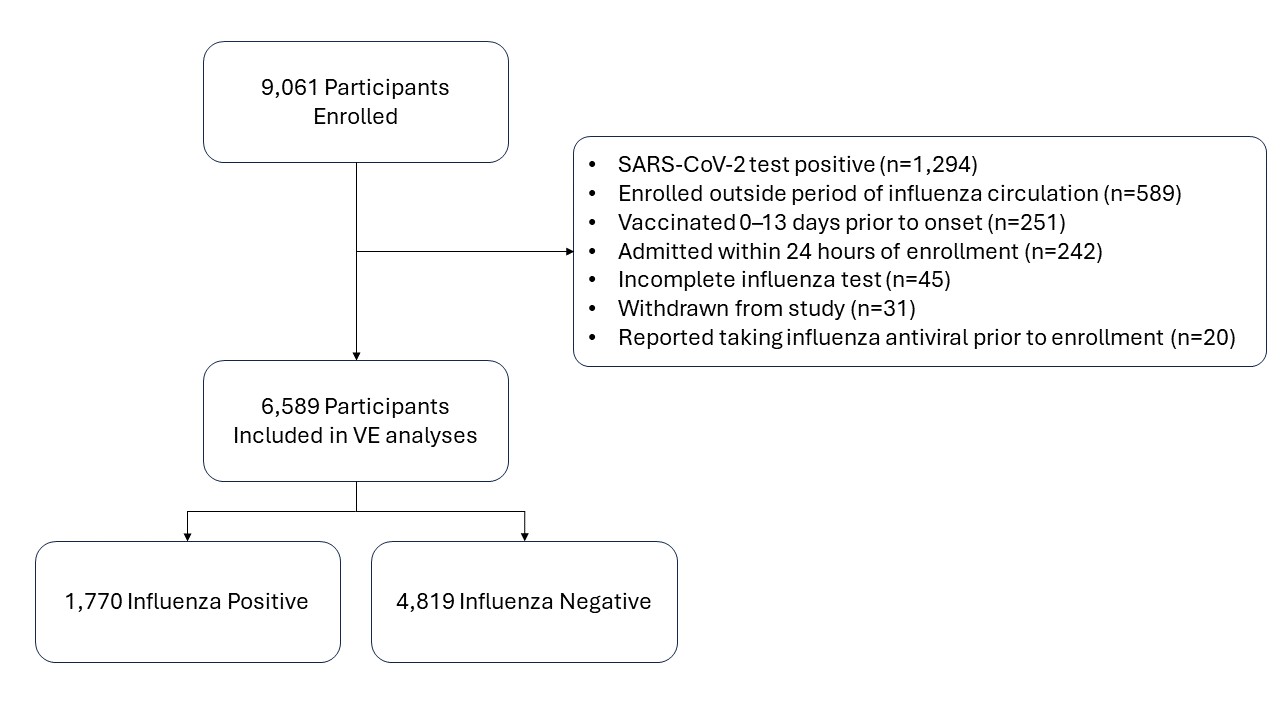


**Supplemental Figure 2.** Age group distribution of influenza A(H1N1)pdm09, A(H3N2), B/Victoria cases and influenza-negative control participants, October 2023 – April 2024.

**Supplemental Figure 3.** Influenza-positive cases by type/subtype and percent influenza positive by week of enrollment, October 2023 – April 2024.

**Supplemental Figure 4.** Sequenced A(H1N1)pdm09 virus genetic subclades by week of illness onset, October 2023 – April 2024

**Supplemental Table 2.** Adjusted vaccine effectiveness against any outpatient influenza-associated illness visits among patients aged ≥8 months enrolled at US Influenza Vaccine Effectiveness Network sites, October 2023 through April 2024 by time since vaccination.

| **Age group** | **Influenza Positive (Cases)** | | **Influenza Negative (Controls)** | | **Vaccine Effectiveness^a^** | |
| --- | --- | --- | --- | --- | --- | --- |
| Time since vaccination (days) | **Total** | **No. Vaccinated (%)** | **Total** | **No. Vaccinated (%)** | **VE %** | **(95% CI)** |
| **All ages ≥8 months** |  |  |  |  |  |  |
| 14–59 | 1486 | 58 (4) | 3566 | 491 (14) | 64 | (52 to 73) |
| 60–120 | 1587 | 159 (10) | 3771 | 696 (18) | 29 | (14 to 42) |
| >120 | 1551 | 123 (8) | 3634 | 559 (15) | 40 | (25 to 52) |
| **8 months –17 years** |  |  |  |  |  |  |
| 14–59 | 575 | 12 (2) | 1086 | 118 (11) | 83^b^ | (70 to 91) |
| 60–120 | 594 | 31 (5) | 1094 | 126 (12) | 51 | (27 to 69) |
| >120 | 582 | 19 (3) | 1070 | 102 (10) | 68^b^ | (48 to 81) |
| **18–64 years** |  |  |  |  |  |  |
| 14–59 | 839 | 36 (4) | 2097 | 257 (12) | 57 | (39 to 71) |
| 60–120 | 895 | 92 (10) | 2184 | 344 (16) | 23 | (0 to 40) |
| >120 | 873 | 70 (8) | 2111 | 271 (13) | 37 | (16 to 53) |
| **≥65 years** |  |  |  |  |  |  |
| 14–59 | 72 | 10 (14) | 383 | 116 (30) | 63^b^ | (28 to 83) |
| 60–120 | 98 | 36 (37) | 493 | 226 (46) | 15 | (-37 to 48) |
| >120 | 96 | 34 (35) | 453 | 186 (41) | 18 | (-35 to 51) |

Abbreviations: CI, confidence interval; VE, vaccine effectiveness

^a^ Models adjusted for study site, age, presence of ≥1 underlying health condition, and month of illness onset. 95% confidence intervals that exclude 0% are considered statistically significant

^b^ Unadjusted due to small sample size

**Supplemental Table 3**. Sensitivity analyses of vaccine effectiveness against any influenza among participants of all ages

|  | **Influenza Positive (Cases)** | | **Influenza Negative (Controls)** | | **Vaccine Effectiveness^a^** | |
| --- | --- | --- | --- | --- | --- | --- |
| **Sensitivity Analysis** | **Total** | **No. Vaccinated (%)** | **Total** | **No. Vaccinated (%)** | **VE %** | **(95% CI)** |
| **Primary analysis** | 1770 | 466 (26) | 4819 | 2023 (42) | 41 | (32 to 49) |
|  |  |  |  |  |  |  |
| **Varying exposure information source** |  |  |  |  |  |  |
| Defining vaccination status using self-reported information only | 1693 | 421 (25) | 4461 | 1710 (38) | 32 | (21 to 41) |
| Defining vaccination status using documented information only | 1801 | 340 (19) | 4927 | 1746 (35) | 44 | (35 to 52) |
|  |  |  |  |  |  |  |
| **Varying participant inclusion criteria** |  |  |  |  |  |  |
| Restricting to participants enrolled within 5 days of illness onset | 1605 | 407 (25) | 3937 | 1633 (41) | 41 | (32 to 49) |
| Including SARS-CoV-2-positive participants | 1803 | 473 (26) | 5825 | 2537 (44) | 45 | (37 to 52) |
| Removing participants from Arizona | 1564 | 454 (29) | 4448 | 1961 (44) | 40 | (30 to 47) |
| Removing participants from Michigan | 1688 | 432 (26) | 4534 | 1874 (41) | 42 | (33 to 49) |
| Removing participants from Missouri | 1425 | 379 (27) | 4385 | 1885 (43) | 45 | (37 to 53) |
| Removing participants from Ohio | 1513 | 405 (27) | 4310 | 1855 (43) | 43 | (34 to 51) |
| Removing participants from Pennsylvania | 1417 | 360 (25) | 3994 | 1617 (40) | 37 | (26 to 46) |
| Removing participants from Texas | 1419 | 367 (26) | 3372 | 1383 (41) | 38 | (28 to 47) |
| Removing participants from Washington | 1594 | 399 (25) | 3871 | 1563 (40) | 42 | (33 to 50) |

^a^ Models adjusted for study site, age, presence of ≥1 underlying health condition, and month of illness onset. 95% confidence intervals that exclude 0% are considered statistically significant
